# Morning versus evening dosing of desloratadine in seasonal allergic rhinitis: a randomized controlled study [ISRCTN23032971].

**DOI:** 10.1186/1476-7961-3-3

**Published:** 2005-02-02

**Authors:** Rolf Haye, Kjetil Høye, Olof Berg, Sissel Frønes, Tone Ødegård

**Affiliations:** 1 Rikshospitalet, 0027 Oslo, Norway; 2 Helsetorget Legesenter, 2408 Elverum, Norway; 3 Betania, ENT-clinic, 114 38 Stockholm, Sweden; 4 Schering-Plough AS, 1359 Eiksmarka, Norway

## Abstract

**Background:**

A circadian rhythm of symptoms has been reported in allergic rhinitis and some studies have shown the dosing time of antihistamines to be of importance for optimizing symptom relief in this disease. The objective of this study was to examine the efficacy of morning vs. evening dosing of the antihistamine desloratadine at different time points during the day.

**Methods:**

Patients ≥ 18 years, with seasonal allergic rhinitis received desloratadine 5 mg orally once daily in the morning (AM-group) or evening (PM-group) for two weeks. Rhinorrhea, nasal congestion, sneezing and eye symptoms were scored morning and evening. Wilcoxon rank sum and 2-way ANOVA test were used.

**Results:**

Six-hundred and sixty-three patients were randomized; 336 in the AM-group; 327 in the PM-group. No statistically significant differences were seen between the AM and PM group at any time points. In the sub-groups with higher morning or evening total symptom score no difference in treatment efficacy was seen whether the dose was taken 12 or 24 hours before the higher score time. There was a circadian variation in baseline total symptom score; highest during daytime and lowest at night. The circadian variation in symptoms was reduced during treatment. This reduction was highest for daytime symptoms.

**Conclusions:**

A circadian rhythm was seen for most symptoms being more pronounced during daytime. This was less apparent after treatment with desloratadine. No statistically significant difference in efficacy was seen whether desloratadine was given in the morning or in the evening. This gives the patients more flexibility in choosing dosing time.

## Background

Allergic rhinitis is a common illness, which affects approximately 15 % of the population [1] and has a large impact on the quality of life of the patients. In some studies the symptoms of allergic rhinitis have shown a circadian rhythm with morning symptoms being most prominent in a majority of patients [1-6].

Antihistamines are important medications in the treatment of allergic rhinitis. One should expect that the effect of an antihistamine is best near or shortly after peak serum level is attained. If this also coincides with the peak in allergy symptoms, an optimal treatment effect should be expected. In one study evening dosing of the antihistamine mequitazine (half-life of 38–45 hours and time to peak serum level about 6 hours) gave better symptom relief than morning dosing on morning symptoms [4,7]. Desloratadine has as mequitazine a rather long half life of 27 hours, and the time to peak serum level at about 3 hours [8]. Evening dosing of this antihistamine may be expected to give better symptom relief than morning dosing on peak morning symptoms. Some studies have also confirmed a circadian variation in efficacy of some antihistamines on histamine induced skin reactions [9,10].

The aim of this study was to examine the efficacy of the antihistamine desloratadine at different time points during the day and to evaluate whether the time of dosing of desloratadine has any impact on the treatment efficacy in seasonal allergic rhinitis (SAR).

## Methods

This was a randomized, open label, parallel group, multicenter study of two weeks duration in patients with SAR during the birch or grass pollen season. Eighty one medical centers in the Nordic countries participated. The inclusion criteria were: patients 18 years or above with a minimum of two years history of SAR confirmed by either a positive skin prick test or a positive serologic allergen test to the relevant seasonal allergen; clinically symptomatic with SAR at baseline/inclusion with a minimum total nasal symptom score (rhinorrhea, congestion, itching and sneezing) of at least 6 and rhinorrhea being minimum 2 (moderate); willingness to adhere to dosing and visit schedule. Females of childbearing potential had to use medically accepted methods of birth control and written informed consent had to be obtained from all patients.

The exclusion criteria were: pulmonary disease, perennial rhinitis, sinusitis, rhinitis medicamentosa, pollen desensitization during the last 6 months, respiratory tract infection within the last two weeks, structural nasal abnormalities (including polyps), use of oral, nasal, ocular decongestants, corticosteroids in any form (except mild dermatological group I corticosteroids allowed in only small areas), other antihistamines (oral or topical), any investigational drug during the last 30 days, pregnant or nursing females.

The patients were randomized into one of two treatment groups with dosing of 5 mg desloratadine tablets either in the morning between 07 – 09 (AM-group) or evening between 19 – 21 (PM-group) in a 1:1 ratio. Randomizing was computer generated for the whole study population using SAS version 6.12 and performed in blocks of eight. Each subject unit (bottle with medication) was labelled with randomization number. Physicians in the different Nordic countries recruited the patients. They assigned the medication in consecutive order. The study was monitored by Schering-Plough.

The following symptoms were assessed using a scale from 0 to 3 (0=none, 1=mild, 2=moderate, 3=severe): rhinorrhea, nasal congestion, sneezing, itching nose and eye symptoms (itching, burning, tearing, redness). These symptoms were recorded in a patient diary every morning (AM 12 hours reflective and AM last hour) and evening (PM 12 hours reflective and PM last hour) both at baseline and during the 2 weeks treatment period. Interference with sleep and interference with daily activity were also assessed by the patients every day using the same scale from 0 to 3. In addition, the number of hours spent outdoors was recorded.

Visit 1 was at day 0 at the start of baseline, visit 2 after one week and visit 3 after two weeks. A wash-out period prior to Visit 1 was necessary if the patient had been on any drugs which could interfere with the study results (e.g. no other commonly used antihistamines allowed during the prior 10 days). Baseline symptoms were recorded in the evening at day 0 and the following morning (day 1) after which the patients started taking the study medication as randomized. A physical examination was performed at visit 1 and 3. All adverse events were recorded. The study period was from April 11^th ^2001 to September 2^nd ^2002. Pollen counts were not recorded.

The primary objective was to evaluate the efficacy of 5 mg desloratadine taken orally once daily in the morning versus evening. The primary efficacy variable was the mean change from baseline for the AM last hour Total Symptom Score (TSS) over the 2 weeks treatment period. TSS is the sum of the individual symptom scores for the following symptoms that in prior studies [2,3] have shown a circadian rhythm: rhinorrhea, nasal stuffiness/congestion, sneezing and eye symptoms (maximum score 12). Since nasal itching had shown little circadian rhythm in these studies, this symptom was omitted from the TSS. AM last hour was chosen as primary time point since the symptoms had in the same studies shown to be worst in the morning. The study was designed to enrol 700 patients in order to have 600 evaluable patients. This sample size was chosen to detect with 90 % power and 5 % significance level, a difference between treatment groups of 0.6 units or more in mean change from baseline diary TSS, assuming a pooled SD of 2.25 units.

In a study on morning vs. evening dosing of the antihistamine mequitazine the differences in dosing-time-related efficacy increased in the sub-group of patients having predominantly morning symptoms [4]. A sub-group analysis was therefore performed on patients with a higher TSS (≥ 1 point difference at baseline) in the morning (AM last hour) than in the evening (PM last hour) and patients with higher TSS in the evening than in the morning in this study. A comparison was then done on the treatment efficacy seen 12 hours and 24 hours after dosing (AM vs. PM dosing) in these patients.

All patients receiving at least one dose of study drug and having at least one post dose registration were included in the efficacy analysis (intention-to-treat, ITT), and confirmatory analysis were based on evaluable patients with no protocol violations. Statistical analyses were made with 2-way ANOVA. For evaluation of response of therapy Wilcoxon rank sum test was used. Adverse events were tabulated.

The study protocol and the patient informed consent form were approved by Ethics Committees and Health Authorities in each of the participating countries.

## Results

### Patients

**Figure 1:**
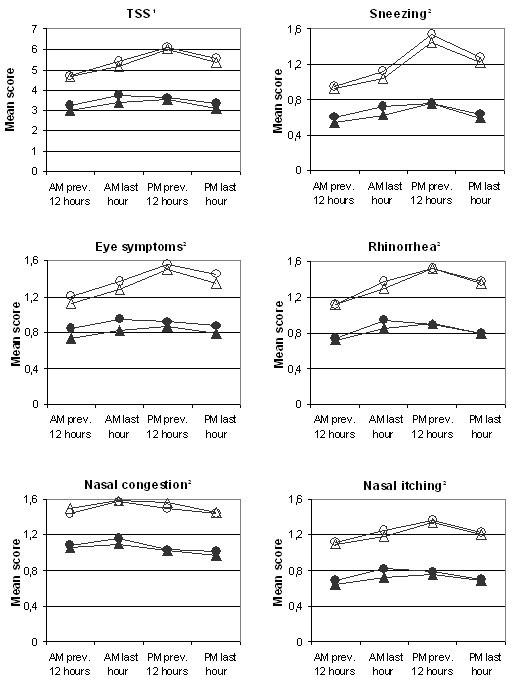
**Symptom scores. **Total and individual symptom scores at baseline and over two weeks treatment period **Baseline: **○ AM-group; Δ PM-group, **Treatment: **● AM-group; ▲ PM-group ^1 ^Max score = 12, ^2 ^Max score = 3

Six hundred and sixty-three patients were randomized at baseline; 336 to the AM-group and 327 to the PM-group. The two groups were comparable with respect to demographics and baseline characteristics (Tab 1). To assess the primary parameter 310 in the AM and 294 in the PM group fulfilled the criteria for ITT. Of the AM group 259 and of the PM group 254 patients completed the study without any violation. Mean baseline TSS varied between 4.64 and 6.10, a difference of 31%; highest during daytime (PM 12 hours reflective) and lowest at night (AM 12 hours reflective). The circadian variation at baseline was more evident for sneezing (around 60% difference between night and day), rhinorrhea and eye symptoms, less so for nasal itching and hardly noticeable for nasal congestion. Fig. 1 shows total and individual symptom scores at baseline and during two weeks treatment. The circadian variation was much less apparent during treatment with desloratadine (Fig. 1).

### Efficacy

During the two weeks period the mean reduction in TSS ± SE for AM last hour (primary efficacy variable) was 1.63 ± 0.17 (30 %) for the AM-group and 1.80 ± 0.17 (35 %) for the PM-group. There was no statistically significant difference (ITT-analysis) between the groups at this time point (p = 0.456) or at any other time points. The reduction in TSS was highest (2.5 – 41%) for day time symptoms (PM previous 12 hours) and lowest at night. This was evident for all individual symptoms except for nasal congestion.

**Figure 2:**
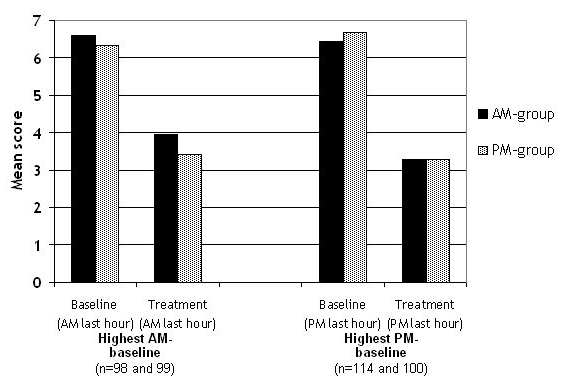
**Sub-group Total Symptom Score. **These sub-groups consists of patients with higher (one or more score points) morning TSS (AM last hour) than evening TSS (PM last hour) at baseline and of patients with higher evening TSS (PM last hour) than morning TSS (AM last hour) at baseline. There was no statistical significant difference in the treatment efficacy between the AM-group and the PM-group.

In the subgroup analysis comparing TSS AM last hour and PM last hour at baseline, 32 % of the patients had more severe symptoms in the morning (≥ 1 point difference in TSS) than in the evening, and 37 % had more severe symptoms in the evening. Looking at these two sub-groups, no difference in treatment efficacy on TSS was seen 12 or 24 hours post dosing (Fig. 2).

**Figure 3:**
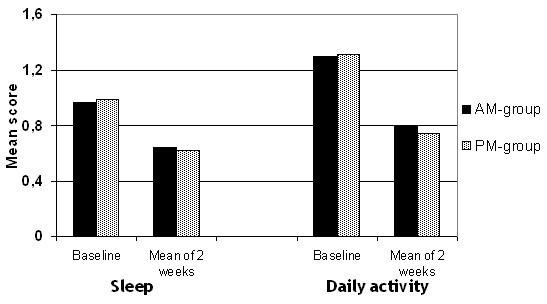
**Sleep and daily activity. **The score for interference with sleep and daily activity at baseline and during treatment shows that there is a higher interference with daily activity at baseline and during treatment than with sleep.

According to their diaries the patients spent in average more than 3.5 hours outdoors daily. The score for the interference of SAR on the patients' sleep and daily activity at baseline and throughout the study is shown in Fig. 3.

### Safety

The incidence of treatment related adverse events were comparable between the groups, 20 % in the AM-group and 18 % in the PM-group, headache being most frequent, 7 % and 4 % respectively.

## Discussion

This study was randomized but without a placebo control. Since this study was a comparison between two different dosing times of the same medication, a placebo control was superfluous. The study was not blinded as there is no reason to believe that neither the patients nor the physicians should have a biased opinion as to the time of dosing. To blind such a study, the patients need to take study medication from different boxes in the morning and evening. However, this method was not used since this may complicate the study and impair patient compliance.

A circadian rhythm has been found in many diseases, also in allergic rhinitis [1-6]. The effect of an antihistamine may be modulated [9-13] by variations in allergen exposure, hormonal activity, organ sensitivity and plasma concentration of the drug. In this study we have shown that desloratadine maintains its effect at different time points throughout the day and thus the effect appears unaffected by a modulating factor.

The baseline period in this study lasted 24 hours which is the same as in the study on mequitazine [4] and other studies [16,17]. In some studies of the effect of antihistamines the baseline period has been longer [14,15]. It would have been difficult to keep patients in the Nordic countries off medication for more than one day in addition to any washout period during the pollen season. We do not believe that the duration of baseline influenced the results of this comparative study.

The circadian rhythm at baseline found in this study with maximum symptoms during the day differs from some other studies [1-6] where more patients had the most severe symptoms in the morning. This difference may partly be due to patient selection. Patients with perennial rhinitis were excluded from our study. Thus indoor allergens do not influence symptom variation. The patients spent several hours outdoors during the day in the pollen season. It seems likely that this exposure would influence the symptoms. The circadian variation was not apparent during treatment, possibly because the suppression of symptoms by desloratadine is more observable when symptoms are most prominent.

The best effect of mequitazine was obtained after evening dosing (12 hours before peak of symptoms) compared to morning dosing (24 hours before peak of symptoms). In our study no difference in treatment efficacy was seen 12 or 24 hours after dosing in the sub-group analysis of patients with higher baseline morning or evening TSS. Whatever the cause for this discrepancy between these two antihistamines, other antihistamines may show a variation in effect during the day not only on dermal symptoms [9,12] but also on nasal ones. Thus studies on the effect of other antihistamines in allergic rhinitis should be encouraged.

The adverse events recorded were of a magnitude and nature as seen in other studies of desloratadine and other antihistamines [14-17].

Many patients have circadian variations in symptoms. The peak of symptoms can be at different time points from patient to patient. Individual dosing time of medication may improve symptom relief. Desloratadine, however, apparently shows no circadian variation in effect.

## Conclusions

A circadian rhythm was seen for most SAR symptoms at baseline, being most distressing during daytime, possibly due to long outdoor exposure. This circadian variation is less apparent after treatment with desloratadine. No statistically significant difference in efficacy was seen whether desloratadine was given in the morning or in the evening. This gives the patients more flexibility in choosing dosing time.

## Competing interests

The study was funded by Schering-Plough in the Nordic countries.

None of the authors will gain financially from the publication. There are no patents pending. There are no other competing interests.

## Authors' contributions

RH participated in the design of the protocol and is the main author of the article.

KH participated in the design of the protocol and as investigator.

OB participated as principal investigator in Sweden and enrolled most patients in the study.

SF participated in the statistical analysis, drafted the tables and figures and participated in drafting the manuscript.

TØ was project leader and participated in the design of the protocol, the statistical analysis and drafting of the manuscript.

**Table 1:** Demographics

	**Treatment groups**		
		
**Demographic Characteristics**	AM-group (n = 336)	PM-group (n = 327)	p-value
**Age (years)**			
Mean (SD)	35.4 (11.0)	36.5 (12.0)	0.232^a^
Min-Max	18–69	18–75	
			
**Age Group (years)**			
18–29	115 (34.2 %)	113 (34.6 %)	
30–39	113 (33.6 %)	95 (29.1 %)	
40–49	70 (20.8 %)	66 (20.2 %)	
≥ 50	38 (11.3 %)	53 (16.2 %)	
			
**Sex**			
Male (%)	152 (45)	163 (50)	0.235^b^
Female (%)	184 (55)	164 (50)	
**Duration of SAR History (years)**			
			
Mean (SD)	14.2 (9.2)	14.4 (10.3)	0.662^a^
Min-Max	2–50	1–55^c^	

^a ^t-test for differences between the means of the two treatment groups

^b ^χ^2^-test for the distribution between the two groups

^c ^One patient in the PM-group had only one year duration of SAR history although the inclusion criterion was 2 years
